# Supplementary material for: Characterisation of a nucleo-adhesome
Source: Nat Commun. 2022 Jun 1;13:3053. doi: 10.1038/s41467-022-30556-5 (PMC9160004; doi:10.1038/s41467-022-30556-5)
Supplement: Supplementary file 3 — Description of Additional Supplementary Files [file 41467_2022_30556_MOESM3_ESM.docx]

**Description of Additional Supplementary Files**

File Name: Supplementary Data 1

Description: Functional enrichment analysis of the meta-adhesome

File Name: Supplementary Data 2

Description: Subcellular proteomic analysis of SCC cells

File Name: Supplementary Data 3

Description: Nuclear meta-adhesome and literature-curated adhesome proteins in SCC cells

File Name: Supplementary Data 4

Description: Functional enrichment analysis of the high-stringency SCC cell nucleo-adhesome

File Name: Supplementary Data 5

Description: Nuclear proteomes of SCC FAK−/−, FAK-WT and FAK-NLS cells

File Name: Supplementary Data 6

Description: FAK-dependent nuclear proteome of SCC cells

File Name: Supplementary Data 7

Description: Gene Ontology enrichment analysis of the FAK-dependent nuclear proteome

File Name: Supplementary Data 8

Description: Transcriptomic analysis of FAK-dependent nuclear proteins

File Name: Supplementary Data 9

Description: Correlation analysis of FAK-dependent nuclear multi-omic data

File Name: Supplementary Data 10

Description: FAK-proximal nuclear proteome of SCC cells

File Name: Supplementary Data 11

Description: Hic-5-proximal nuclear proteome of SCC cells

File Name: Supplementary Data 12

Description: Curated subcellular marker proteins
