# Supplementary material for: Characterisation of a nucleo-adhesome
Source: Nat Commun. 2022 Jun 1;13:3053. doi: 10.1038/s41467-022-30556-5 (PMC9160004; doi:10.1038/s41467-022-30556-5)
Supplement: Supplementary file 16 — Reporting Summary [file 41467_2022_30556_MOESM16_ESM.pdf]

nature portfolio

Corresponding author(s): Adam Byron

Last updated by author(s): Apr 29, 2022

Reporting Summary

Nature Portfolio wishes to improve the reproducibility of the work that we publish. This form provides structure for consistency and transparency in reporting. For further information on Nature Portfolio policies, see our Editorial Policies and the Editorial Policy Checklist.

Statistics

For all statistical analyses, confirm that the following items are present in the figure legend, table legend, main text, or Methods section.

n/a	Confirmed	
☐	☒	The exact sample size (*n*) for each experimental group/condition, given as a discrete number and unit of measurement
☐	☒	A statement on whether measurements were taken from distinct samples or whether the same sample was measured repeatedly
☐	☒	The statistical test(s) used AND whether they are one- or two-sided<br>*Only common tests should be described solely by name; describe more complex techniques in the Methods section.*
☒	☐	A description of all covariates tested
☐	☒	A description of any assumptions or corrections, such as tests of normality and adjustment for multiple comparisons
☐	☒	A full description of the statistical parameters including central tendency (e.g. means) or other basic estimates (e.g. regression coefficient) AND variation (e.g. standard deviation) or associated estimates of uncertainty (e.g. confidence intervals)
☐	☒	For null hypothesis testing, the test statistic (e.g. *F*, *t*, *r*) with confidence intervals, effect sizes, degrees of freedom and *P* value noted<br>*Give P values as exact values whenever suitable.*
☒	☐	For Bayesian analysis, information on the choice of priors and Markov chain Monte Carlo settings
☒	☐	For hierarchical and complex designs, identification of the appropriate level for tests and full reporting of outcomes
☒	☐	Estimates of effect sizes (e.g. Cohen's *d*, Pearson's *r*), indicating how they were calculated

Our web collection on statistics for biologists contains articles on many of the points above.

Software and code

Policy information about availability of computer code

Data collection	Image Lab (version 5.2.1, Bio-Rad).
Data analysis	Image Lab (version 5.2.1, Bio-Rad); Fiji (version 1.53h); MaxQuant (version 1.6.2.10); imp4p (version 1.0); impute (version 1.64.0); Perseus (version 1.5.2.6); VennMaster (version 0.38.2); AmiGO 2 (version 2.5.12); WebGestalt (version 2019); ToppGene (version 2019-Oct-08 21:31); g:Profiler (versions e98_eg45_p14_ce5b097 and e101_eg48_p14_baf17f0); GeneMANIA (versions 3.5.1 and 3.5.2); Cytoscape (version 3.7.1); EnrichmentMap (version 3.2.1); kallisto (version 0.43.1); DESeq2 (version 1.24.0); tximport (version 1.2.0); MSnbase (version 2.15.7); pRoloc (version 1.30.0); Cluster 3.0 (C Clustering Library, version 1.54); Java TreeView (version 1.1.5r2); DeepLoc (version 1.0); Excel (version 16.16.23, Microsoft); PlotsOfData (version 1.0.5); Prism (version 8.3.0, GraphPad); SuperPlotsOfData (version 1.0.3).

For manuscripts utilizing custom algorithms or software that are central to the research but not yet described in published literature, software must be made available to editors and reviewers. We strongly encourage code deposition in a community repository (e.g. GitHub). See the Nature Portfolio guidelines for submitting code & software for further information.

Data

Policy information about availability of data

All manuscripts must include a data availability statement. This statement should provide the following information, where applicable:

- Accession codes, unique identifiers, or web links for publicly available datasets
- A description of any restrictions on data availability
- For clinical datasets or third party data, please ensure that the statement adheres to our policy

MS data that support the findings of this study have been deposited in ProteomeXchange via the PRIDE partner repository with the dataset accession identifiers PXD025870 [http://proteomecentral.proteomexchange.org/cgi/GetDataset?ID=PXD025870] (subcellular fractionation profiling), PXD020179 [http://

proteomecentral.proteomexchange.org/cgi/GetDataset?ID=PXD020179] (FAK-dependent nuclear subproteome), PXD025861 [http://proteomecentral.proteomexchange.org/cgi/GetDataset?ID=PXD025861] (FAK-proximal nuclear interactome) and PXD025868 [http://proteomecentral.proteomexchange.org/cgi/GetDataset?ID=PXD025868] (Hic-5-proximal nuclear interactome). RNA-Seq data that support the findings of this study have been deposited in the Gene Expression Omnibus with GEO series accession identifier GSE147670 [https://www.ncbi.nlm.nih.gov/geo/query/acc.cgi?acc=GSE147670]. Protein sequence and functional annotation data used in this study are available in the UniProtKB database version 2018_07 [https://ftp.uniprot.org/pub/databases/uniprot/previous_releases/release-2018_07/] and version 2021_02 [https://ftp.uniprot.org/pub/databases/uniprot/previous_releases/release-2021_02/], respectively; protein subcellular localisation data used in this study are available in the Human Protein Atlas version 20.1 [https://v20.proteinatlas.org]. All other data supporting the findings of this study are available within the paper and its Supplementary Information and Source Data files. Source data are provided with this paper.

Field-specific reporting

Please select the one below that is the best fit for your research. If you are not sure, read the appropriate sections before making your selection.

[x] Life sciences [ ] Behavioural & social sciences [ ] Ecological, evolutionary & environmental sciences

For a reference copy of the document with all sections, see nature.com/documents/nr-reporting-summary-flat.pdf

Life sciences study design

All studies must disclose on these points even when the disclosure is negative.

Sample size	Sample size was not predetermined in this study. The number of independent experimental samples used for each experiment is indicated in the Methods section or corresponding figure legend. Sample sizes of 3–5 independent biological replicates and, for image quantification, greater than 50 cells per experimental group were determined empirically from previous experimental evidence with similar assays to be sufficient to detect minimal biologically relevant differences between experimental conditions at the 5% significance level (Canel et al. (2017) Cancer Res. 77, 5301–5312; Serrels et al. (2017) Sci. Signal. 10, eaan8355; Schoenherr et al. (2020) J. Biol. Chem. 295, 12045–12057; Acebrón et al. (2020) EMBO J. 39, e104743; Griffith et al. (2021) Sci. Rep. 11, 229; Rabanal-Ruiz et al. (2021) J. Cell Biol. 220, e202004010; Gangoso et al. (2021) Cell 184, 2454–2470; Beetham et al. (2022) Cancer Res. 82, 632–647).
Data exclusions	For MS data analysis, proteins that were not quantified in at least four out of five, at least three out of four or three out of three independent biological replicates for at least one experimental condition were excluded from further analysis to minimise missing values, improve missing value imputation across the dataset and improve accuracy of quantification. For RNA-Seq data analysis, following summarisation of transcript abundance to gene level, genes with zero read counts were removed prior to differential expression analysis, a minimal filtering step to remove features with no information about gene expression. For multi-omic analysis, only features quantified by both proteomics and transcriptomics were integrated to enable complete coverage of the dataset.
Replication	Experimental findings were replicated using multiple independent biological replicates as indicated in the Methods section or corresponding figure legend.
Randomization	Randomisation of sample allocation was not relevant to the experiments in this study (there were no animal or clinical case–control experiments). For comparative experiments in vitro, the same number of cells were randomly apportioned to each sample.
Blinding	Experiments in this study were performed non-blinded to experimental group allocation as complicated experimental designs rendered blinding unfeasible. For computational analyses, experimental group allocation was not provided to the algorithms.

Reporting for specific materials, systems and methods

We require information from authors about some types of materials, experimental systems and methods used in many studies. Here, indicate whether each material, system or method listed is relevant to your study. If you are not sure if a list item applies to your research, read the appropriate section before selecting a response.

Materials & experimental systems

n/a	Involved in the study	
[ ]	[x]	Antibodies
[ ]	[x]	Eukaryotic cell lines
[x]	[ ]	Palaeontology and archaeology
[ ]	[x]	Animals and other organisms
[x]	[ ]	Human research participants
[x]	[ ]	Clinical data
[x]	[ ]	Dual use research of concern

Methods

n/a	Involved in the study	
[x]	[ ]	ChIP-seq
[x]	[ ]	Flow cytometry
[x]	[ ]	MRI-based neuroimaging

Antibodies

Antibodies used	The following antibodies were used for immunoblotting (all Cell Signaling Technology, diluted 1:1,000, unless otherwise stated): α-tubulin (#3873, diluted 1:2,000), GAPDH (#2118; #5174), ERp72 (#5033), RCAS1 (#12290), GM130 (#610822, BD Biosciences), Sun2

(#ab87036, Abcam), lamin-A/C (#4777), PARP1 (#9532), histone H4 (#2935), AFAP1 (#610200, BD Biosciences), c-Met (#4560), APPL1 (#3858), EEA1 (#2411), cathepsin B (#31718), golgin-97 (#13192), testin (#sc-373913, Santa Cruz Biotechnology), FAK (#3285), histone H3 (#4620), HP1α/β (#2623), Hic-5 (#611164, BD Biosciences), HRP-conjugated anti-rabbit IgG (#7074, diluted 1:10,000), HRP-conjugated anti-mouse IgG (#7076, diluted 1:5,000), HRP-conjugated streptavidin (#3999). The following antibodies were used for immunofluorescence (all diluted 1:200 unless otherwise stated): Hic-5 (#611164, BD Biosciences, diluted 1:150), paxillin (#612405, BD Biosciences), testin (#sc-271184, Santa Cruz Biotechnology), Alexa Fluor 488-conjugated anti-mouse IgG (#A11001, Invitrogen, diluted 1:400).

Validation

Detailed information on the antibodies used, including validation information and relevant references, is provided via the Resource Identification Portal [https://scicrunch.org/resources] (RRID antibody ID provided in parentheses) and the respective manufacturer's website as follows:
α-tubulin (#3873, Cell Signaling Technology) [https://www.cellsignal.com/products/primary-antibodies/a-tubulin-dm1a-mouse-mab/3873] (AB_1904178) [https://antibodyregistry.org/search.php?q=AB_1904178]
GAPDH (#2118, Cell Signaling Technology) [https://www.cellsignal.com/products/primary-antibodies/gapdh-14c10-rabbit-mab/2118?site-search-type=Products&N=4294956287&Ntt=gapdh+%28%232118%29&fromPage=plp&_requestid=2195518] (AB_561053) [https://antibodyregistry.org/search.php?q=AB_561053]
GAPDH (#5174, Cell Signaling Technology) [https://www.cellsignal.com/products/primary-antibodies/gapdh-d16h11-xp-rabbit-mab/5174] (AB_10622025) [https://antibodyregistry.org/search.php?q=AB_10622025]
ERp72 (#5033, Cell Signaling Technology) [https://www.cellsignal.com/products/primary-antibodies/erp72-d70d12-xp-rabbit-mab/5033] (AB_10622112) [https://antibodyregistry.org/search.php?q=AB_10622112]
RCAS1 (#12290, Cell Signaling Technology) [https://www.cellsignal.com/products/primary-antibodies/rcas1-d2b6n-xp-rabbit-mab/12290] (AB_2736985) [https://antibodyregistry.org/search.php?q=AB_2736985]
GM130 (#610822, BD Biosciences) [https://www.bdbiosciences.com/en-gb/products/reagents/microscopy-imaging-reagents/immunofluorescence-reagents/purified-mouse-anti-gm130.610822] (AB_398141) [https://antibodyregistry.org/search.php?q=AB_398141]
Sun2 (#ab87036, Abcam) [https://www.abcam.com/sun2-antibody-ab87036.html] (AB_1952674) [https://antibodyregistry.org/search.php?q=AB_1952674]
Lamin-A/C (#4777, Cell Signaling Technology) [https://www.cellsignal.com/products/primary-antibodies/lamin-a-c-4c11-mouse-mab/4777] (AB_10545756) [https://antibodyregistry.org/search.php?q=AB_10545756]
PARP1 (#9532, Cell Signaling Technology) [https://www.cellsignal.com/products/primary-antibodies/parp-46d11-rabbit-mab/9532?site-search-type=Products&N=4294956287&Ntt=parp1+%28%239532%29&fromPage=plp&_requestid=2197102] (AB_659884) [https://antibodyregistry.org/search.php?q=AB_659884]
Histone H4 (#2935, Cell Signaling Technology) [https://www.cellsignal.com/products/primary-antibodies/histone-h4-l64c1-mouse-mab/2935?site-search-type=Products&N=4294956287&Ntt=histone+h4+%28%232935%29&fromPage=plp&_requestid=2197247] (AB_1147658) [https://antibodyregistry.org/search.php?q=AB_1147658]
AFAP1 (#610200, BD Biosciences) (AB_397599) [https://antibodyregistry.org/search.php?q=AB_397599]
c-Met (#4560, Cell Signaling Technology) [https://www.cellsignal.com/products/primary-antibodies/met-antibody/4560?site-search-type=Products&N=4294956287&Ntt=c-met+%28%234560%29&fromPage=plp&_requestid=2197630] (AB_2143887) [https://antibodyregistry.org/search.php?q=AB_2143887]
APPL1 (#3858, Cell Signaling Technology) [https://www.cellsignal.com/products/primary-antibodies/appl1-d83h4-xp-rabbit-mab/3858?site-search-type=Products&N=4294956287&Ntt=appl1+%28%233858%29&fromPage=plp&_requestid=2197769] (AB_2056989) [https://antibodyregistry.org/search.php?q=AB_2056989]
EEA1 (#2411, Cell Signaling Technology) [https://www.cellsignal.com/products/primary-antibodies/eea1-antibody/2411?site-search-type=Products&N=4294956287&Ntt=eea1+%28%232411%29&fromPage=plp&_requestid=2197910] (AB_2096814) [https://antibodyregistry.org/search.php?q=AB_2096814]
Cathepsin B (#31718, Cell Signaling Technology) [https://www.cellsignal.com/products/primary-antibodies/cathepsin-b-d1c7y-xp-rabbit-mab/31718?site-search-type=Products&N=4294956287&Ntt=cathepsin+b+%28%2331718%29&fromPage=plp&_requestid=2198049] (AB_2687580) [https://antibodyregistry.org/search.php?q=AB_2687580]
Golgin-97 (#13192, Cell Signaling Technology) [https://www.cellsignal.com/products/primary-antibodies/golgin-97-d8p2k-rabbit-mab/13192?site-search-type=Products&N=4294956287&Ntt=golgin-97+%28%2313192%29&fromPage=plp&_requestid=2198215] (AB_2798144) [https://antibodyregistry.org/search.php?q=AB_2798144]
Testin (#sc-373913, Santa Cruz Biotechnology) [https://www.scbt.com/p/tes-antibody-g-5] (AB_10918347) [https://antibodyregistry.org/search.php?q=AB_10918347]
FAK (#3285, Cell Signaling Technology) [https://www.cellsignal.com/products/primary-antibodies/fak-antibody/3285?site-search-type=Products&N=4294956287&Ntt=fak+%28%233285%29&fromPage=plp&_requestid=2198513] (AB_2269034) [https://antibodyregistry.org/search.php?q=AB_2269034]
Histone H3 (#4620, Cell Signaling Technology) [https://www.cellsignal.com/products/primary-antibodies/histone-h3-d2b12-xp-rabbit-mab-chip-formulated/4620?site-search-type=Products&N=4294956287&Ntt=histone+h3+%28%234620%29&fromPage=plp&_requestid=2198675] (AB_1904005) [https://antibodyregistry.org/search.php?q=AB_1904005]
HP1α/β (#2623, Cell Signaling Technology) [https://www.cellsignal.com/products/primary-antibodies/hp1a-b-c7f11-rabbit-mab/2623?site-search-type=Products&N=4294956287&Ntt=hp1α%2Fβ+%28%232623%29%2C&fromPage=plp&_requestid=2198799] (AB_2070981) [https://antibodyregistry.org/search.php?q=AB_2070981]
Hic-5 (#611164, BD Biosciences) [https://www.bdbiosciences.com/en-gb/products/reagents/microscopy-imaging-reagents/immunofluorescence-reagents/purified-mouse-anti-hic5.611164] (AB_398702) [https://antibodyregistry.org/search.php?q=AB_398702]
HRP-conjugated anti-rabbit IgG (#7074, Cell Signaling Technology) [https://www.cellsignal.com/products/secondary-antibodies/anti-rabbit-igg-hrp-linked-antibody/7074?site-search-type=Products&N=4294956287&Ntt=%237074&fromPage=plp&_requestid=2199348] (AB_2099233) [https://antibodyregistry.org/search.php?q=AB_2099233]
HRP-conjugated anti-mouse IgG (#7076, Cell Signaling Technology) [https://www.cellsignal.com/products/secondary-antibodies/anti-mouse-igg-hrp-linked-antibody/7076?site-search-type=Products&N=4294956287&Ntt=%237076%2C+&fromPage=plp&_requestid=2199563] (AB_330924) [https://antibodyregistry.org/search.php?q=AB_330924]
HRP-conjugated streptavidin (#3999, Cell Signaling Technology) [https://www.cellsignal.com/products/wb-ip-reagents/streptavidin-hrp/3999?site-search-type=Products&N=4294956287&Ntt=%233999&fromPage=plp&_requestid=2199735] (AB_10830897) [https://

antibodyregistry.org/search.php?q=AB_10830897]
Paxillin (#612405, BD Biosciences) [https://www.bdbiosciences.com/en-gb/products/reagents/microscopy-imaging-reagents/immunofluorescence-reagents/purified-mouse-anti-paxillin.612405] (AB_647289) [https://antibodyregistry.org/search.php?q=AB_647289]
Testin (#sc-271184, Santa Cruz Biotechnology) [https://www.scbt.com/p/tes-antibody-g-9] (AB_10611304) [https://antibodyregistry.org/search.php?q=AB_10611304]
Alexa Fluor 488-conjugated anti-mouse IgG (#A11001, Invitrogen) [https://www.thermofisher.com/antibody/product/Goat-anti-Mouse-IgG-H-L-Cross-Adsorbed-Secondary-Antibody-Polyclonal/A-11001] (AB_2534069) [https://antibodyregistry.org/search.php?q=AB_2534069]

Eukaryotic cell lines

Policy information about cell lines

Cell line source(s)	The mouse squamous cell carcinoma (SCC) FAK cellular model and the FAK-NLS mutant were generated as previously described by our laboratory (see Serrels et al. (2012) Int. J. Cancer 131, 287–297; Serrels et al. (2015) Cell 163, 160–173).
Authentication	None of the cell lines used was authenticated.
Mycoplasma contamination	Cell lines were routinely tested for mycoplasma, tested negative and were used within three months of recovery from frozen.
Commonly misidentified lines (See ICLAC register)	None used.

Animals and other organisms

Policy information about studies involving animals; ARRIVE guidelines recommended for reporting animal research

Laboratory animals	The study did not directly involve laboratory animals. The mouse SCC FAK cellular model was originally generated from female 8-week-old K14CreER-Ptk2flox/flox FVB mice in a previous study (Serrels et al. (2012) Int. J. Cancer 131, 287–297).
Wild animals	The study did not involve wild animals.
Field-collected samples	The study did not involve samples collected from the field.
Ethics oversight	No ethical approval or guidance was required for the study as it did not involve animals. Generation of the SCC FAK cellular model in a previous study (Serrels et al. (2012) Int. J. Cancer 131, 287–297) had University of Glasgow ethical approval and was carried out in accordance with the United Kingdom Animal Scientific Procedures Act (1986) under Home Office Project Licence number 60/4248.

Note that full information on the approval of the study protocol must also be provided in the manuscript.
