# Supplementary material for: Characterisation of a nucleo-adhesome
Source: Nat Commun. 2022 Jun 1;13:3053. doi: 10.1038/s41467-022-30556-5 (PMC9160004; doi:10.1038/s41467-022-30556-5)
Supplement: Supplementary file 17 — Source Data [file 41467_2022_30556_MOESM17_ESM.zip › Source Data.pdf]

Figure 2b

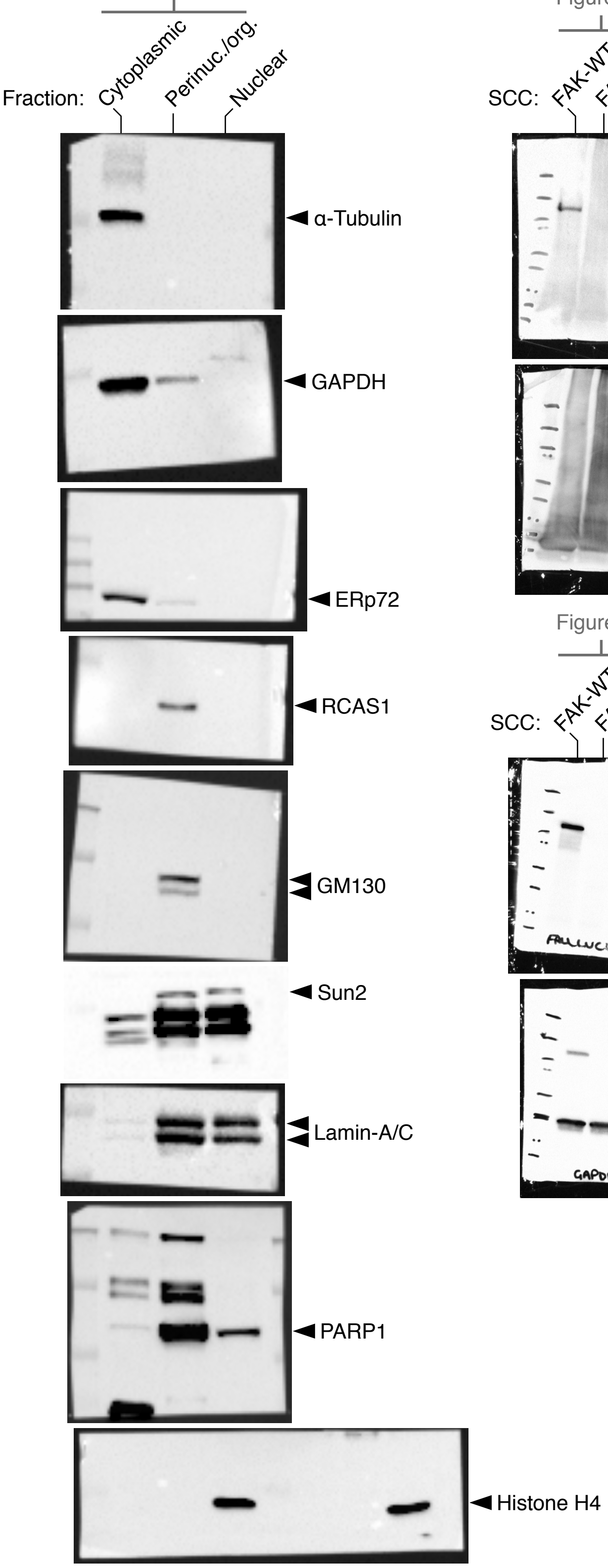

Figure 4a

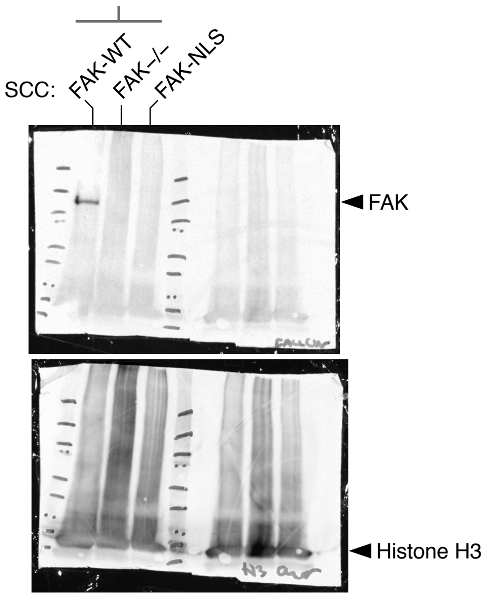

Figure 4b

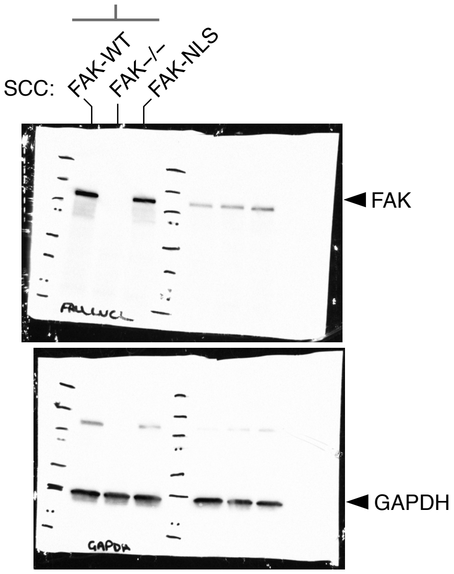

Figure 5h

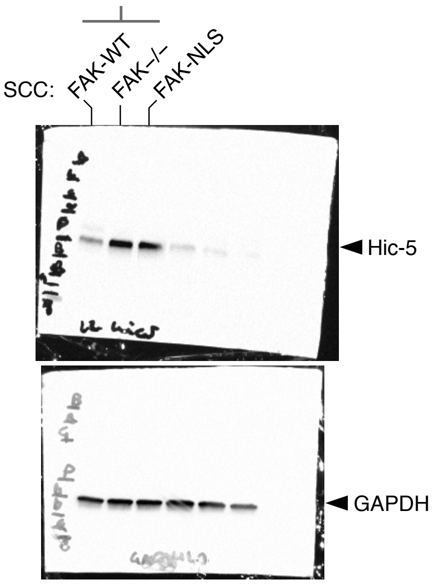

Figure 5i

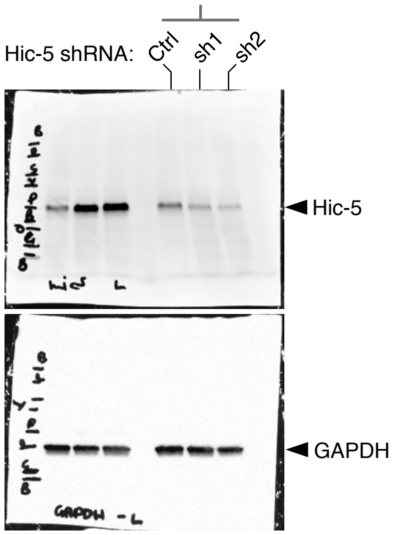

Source Data. Continued on next page.

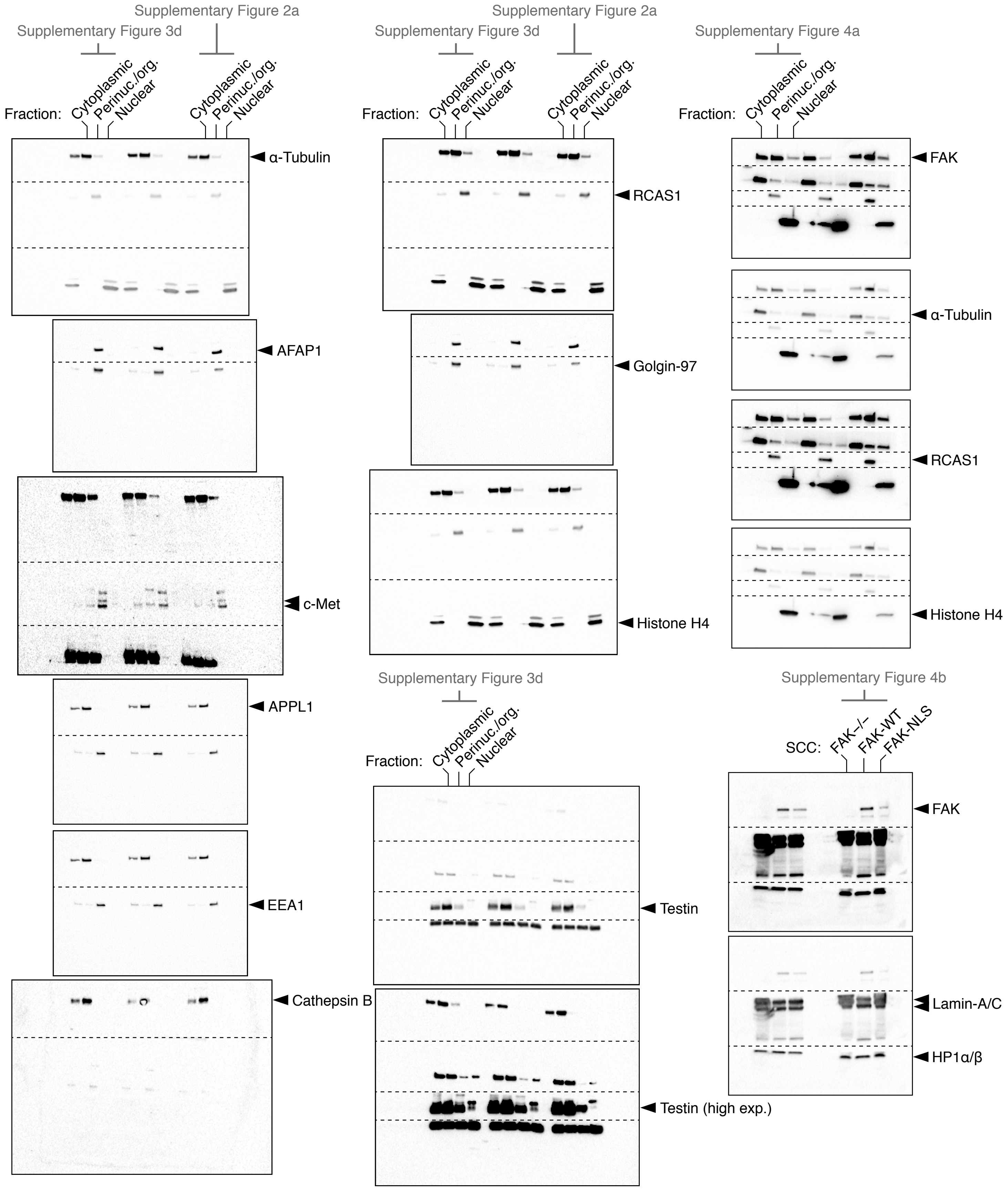

Source Data. Original blots. Arrowheads indicate expected band positions for each protein probed. Dashed lines indicate boundaries between membranes scanned in the same image.
